# Effects of Surface Morphology on Mesoporous Silicon-Modified Nanofiltration Membranes for High Rejection Performances

**DOI:** 10.3390/membranes15090274

**Published:** 2025-09-10

**Authors:** Ying Ding, Aifang Ding, Yuqing Liu, Dong Liu

**Affiliations:** College of Environment Science, Nanjing Xiaozhuang University, Nanjing 211171, China; 2000443@njxzc.edu.cn (A.D.); 18112510208@163.com (Y.L.); 19551172282@163.com (D.L.)

**Keywords:** mesoporous silicon, modified, NF membranes, surface morphology

## Abstract

A novel approach was developed in this work in which composite nanofiltration (NF) membranes were directly and efficiently fabricated with control of the membrane pore structure and surface morphology. The fabrication of mesoporous silicon-modified polysulfone blend membranes is achieved via a phase inversion method. The structural morphology, surface functional group analysis, elemental analysis, hydrophilicity, chargeability, and nitrogen pollutant (ammonia nitrogen, nitrate nitrogen, total nitrogen) rejection properties of the modified membranes were found to be dependent on the amount of mesoporous silicon incorporated. The combination of the mesoporous silicon framework layer can not only effectively improve the surface structure of the modified membrane with a narrow pore size distribution but also increase the rejection of nitrogen pollutants compared with pure NF membranes. The mesoporous material interlayer can absorb and store the aqueous amino solution to facilitate the subsequent interfacial polymerization as well as induce changes in the pore radius and surface structure. Compared with pure NF composite membranes, the modified blend membranes exhibit increased water permeation flux as high as 29.09 L m^−2^ h^−1^ at 0.2 MPa. The results show that the optimum doping amount of mesoporous silicon is in the range of 0.5–1.0%. Characterization studies demonstrated that the addition of mesoporous silicon leads to a decreased membrane pore size. Then the retention of nitrogen pollutants was enhanced because of a combination of hydrophilicity enhancement from the carboxylic and hydroxyl functional groups present in their surfaces leading to electrostatic repulsion between functional groups present in the membranes and the nitrogen pollutant molecules.

## 1. Introduction

Nanofiltration (NF) is used as a pretreatment process for seawater reverse osmosis (SWRO) desalination, food processing, biomedical engineering, and chemical separation due to its significant developmental promise [1,2,3]. Its key characteristics include [4,5] the following: (1) distinctive retention efficacy between monovalent and multivalent salts, demonstrating pronounced ion selectivity; (2) molecular weight cutoff thresholds spanning 200–1000 Da, with certain sub-200 Da inorganic ions retained via Donnan exclusion effects; (3) operational pressure requirements maintained within 0.5–2.0 MPa. The separation mechanisms governing NF membranes involve multifaceted processes: electrostatic repulsion (Donnan effect) governs charged solute rejection, while steric hindrance and solution–diffusion dynamics dictate neutral solute retention [6,7,8].

Nevertheless, commercial NF membranes face limitations including inadequate water flux, elevated operational pressures, and suboptimal removal efficiency for natural organic matter [9,10,11]. Nitrogenous pollutants represent a target contaminant category effectively addressable by NF membranes. To augment separation performance, recent methodologies incorporate inorganic nanoparticles (e.g., TiO_2_, ZnO, SiO_2_) as surface modifiers [12,13]. Furthermore, controlling membrane microstructure during non-solvent-induced phase separation (NIPS) proves challenging; consequently, surface functionalization has emerged as a viable strategy for enhancing permeability and rejection rates [14,15]. Mesoporous materials, owing to their ordered pore configurations and electrochemically active surfaces, are increasingly utilized in membrane modification. Despite their potential, challenges such as particle agglomeration and poor interfacial compatibility hinder the precise characterization of pore architecture and surface morphology post-modification. Moreover, the leakage of nanomaterials in mixed-matrix membranes or composite membranes may bring potential toxicity hazards, which raises doubts about their stability and safety [16]. How to prepare modified membranes with strong stability and good uniformity has become a technical challenge. Overcoming this challenge can effectively prevent secondary pollution of nanomaterials. Excessive additive loading particularly induces particle aggregation, compromising membrane efficacy. Contemporary studies confirm that nanoparticle-enhanced NF membranes display superior structural attributes and rejection performance [17]. Thus, optimizing mesoporous material structure and dosage offers substantial potential for NF membrane advancement.

**Figure 1:**
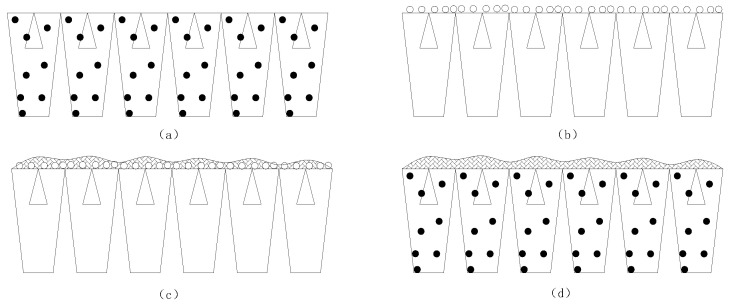
Schematic diagram of modification method of mesoporous materials applied to membrane modification (round particles represent mesoporous materials). (**a**) Blending modification (**b**) Surface immobilization (**c**) Interfacial polymerization (**d**) Substrate compositing.

Recent progress in mesoporous material synthesis has expanded its utility in membrane engineering [18,19]. Attributes including nanoscale dimensions, high specific surface area, and large pore volumes enable integration via blending or surface coating, directly influencing membrane morphology [20]. Four primary modification approaches exist (Figure 1): (a) Blending modification: Incorporation into casting solutions followed by phase inversion, predominantly for ultrafiltration membrane fabrication. (b) Surface immobilization: Physical deposition, chemical grafting, or electrostatic adsorption onto membrane surfaces. (c) Interfacial polymerization: Dispersion within organic phases for composite membrane synthesis. (d) Substrate compositing: Utilization of mesoporous material–casting solution hybrids as support layers. Compared with other modification methods such as surface modification, the blending modification method used in this experiment has high membrane stability and better process adaptability [21,22]. The mesoporous silicon used in this article has better performance, reflected in the strong uniformity of the modified film, more mesoporous adsorption points, and better reactivity than other commonly used microporous materials such as SiO_2_, TiO_2_ [23,24]. Meanwhile, other modified membrane studies have focused on the removal of some large-molecule pollutants. The mesoporous silicon-modified membrane in this article mainly focuses on the separation performance of small-molecule pollutants such as nitrate nitrogen, ammonia nitrogen, and organic nitrogen [25]. To address weak small-molecule pollutant removal, this study introduces mesoporous silica-modified polyethersulfone (PES) composite NF membranes, aiming to refine surface charge distribution and achieve efficient nitrogen pollutant separation. Mesoporous modification alters surface hydrophilicity, zeta potential, and water permeability. Inorganic mesoporous additives further modulate pore size distribution and molecular weight cutoffs, elevating nitrogen pollutant removal efficiency. This work fabricates PES composite NF membranes modified with mesoporous silica, characterizing their fundamental performance parameters. Post-modification, the water permeability significantly increased, while the hydrophobicity and surface charge properties were optimized, facilitating enhanced organic contaminant removal. We systematically investigate additive loading effects via material characterization, compare performance metrics against commercial NF270 membranes, and elucidate the structure–function relationships governing separation enhancement. Compared with traditional commercial nanofiltration membranes NF90 and NF270, the modified membrane in this article exhibits excellent performance in pollutant separation and membrane basic properties such as water flux.

The surface characteristics, such as surface charge, hydrophobicity, and water flux, can be changed by the modification of the mesoporous materials. The addition of inorganic mesoporous materials can also change the pore size and molecular weight of membranes and improve the rejection efficiency of membranes for nitrogen pollutants [26,27,28].

In this study, a PES composite NF membrane was modified by adding mesoporous silica, and the basic performance parameters of the modified PES composite NF membrane were measured. Through the introduction of mesoporous material, the water flux of the PES composite NF membrane was improved, and the hydrophobicity and surface charge characteristics of membranes could be changed, which is beneficial to the removal of organic pollutants by the PES composite NF membrane. In this paper, the amount of modified material is discussed, and the influence of the addition of mesoporous material on the properties of the films is analyzed by means of characterization methods. And we compared the performance index of the PES composite NF membrane with that of commercial NF270 membranes.

## 2. Materials and Methods

### 2.1. Materials

The triblock copolymer Pluronic F127 was obtained from Sigma-Aldrich. Phenol, polyethersulfone (PES), dimethylacetamide (DMAC), ammonium chloride, potassium nitrate, humic acid (HA), and tetraethyl orthosilicate (>98% purity) were purchased from Nanjing chemical reagent Co., Ltd. (Nanjing, China). The NF membranes selected in this study were thin-film composite polyamide membranes, including NF270 (RisingSun Membrane Technology Co., Ltd. Guangzhou, China).

### 2.2. Synthesis of Mesoporous Materials

Mesoporous silica was synthesized according to our previous report [29,30] and other literature [31]. Mesoporous silicon was prepared by adding 4 g of P123 to a mixed solution of hydrochloric acid and deionized water, stirring at 38 °C for more than 2 h until P123 is completely dissolved. Then 8.4 g ethyl orthosilicate (TEOS) was added and stirred for 20 h. The white suspension was poured into a high-pressure reactor to react at 100 °C for 24 h, then rinsed with deionized water and ethanol, filtered, and dried. The material was placed in a porcelain plate and fired at 120 °C for 2 h to remove the template agent from mesoporous silicon.

### 2.3. Fabrication of Mesoporous Material-Modified PES Commercial NF Membranes

The modified PES commercial NF membranes were prepared by phase inversion via the immersion precipitation technique [32]. The casting solution was prepared by mixing 10.0 g of PES with DMAC (40 mL). These components were stirred at 300 rpm and 25 °C for 12 h. Then 0.55 g of lithium chloride (LiCl) and 5.0 g of polyvinylpyrrolidone (PVP) were sequentially added to the reaction mixture prepared in the previous step. These components were stirred at 300 rpm for 24 h. Then the solution was prepared by mixing 1.0 wt% of mesoporous silica and stirring for 6 h. After the casting solution is completely mixed, it removed bubbles by standing still. The commercial nanofiltration membranes were immersed in the solutions, and they were sprinkled and then cast on commercial nanofiltration membranes. The modified films were immersed in a dimethylacetamide solution (5.0 wt%) and deionized water for 5 min each for membrane formation. The prepared membranes were rinsed at least 5 times and stored in deionized water for at least 72 h to completely remove the residual solvents and additives.

Membranes incorporating mesoporous silicon were fabricated in a similar fashion. The different compositions of modified PES membranes are shown in Table 1.

### 2.4. Membrane Characterization

#### 2.4.1. Static Contact Angle Test (SCA)

The surface hydrophilicity of membranes was characterized using a contact angle goniometer (Model: OCA20, DataPhysics, Beijing, China). A water droplet (3 μL) was deposited onto each membrane surface through a needle tip attached to the goniometer. A magnified image of the water droplet was observed by a digital camera, and the CA readings were obtained after 10 s of water drop. The contact angles of each membrane at 5 different locations were recorded, and their mean values were calculated.

#### 2.4.2. Zeta Potential Tests

To study the surface charge properties of the modified NF membranes, the ζ-potential of the pure NF membrane and modified NF membrane surfaces as a function of pH from 2.5 to 10 was analyzed by a SurPASS electrokinetic analyzer. The ζ-potential of the membrane was then measured accordingly at different pH values.

#### 2.4.3. XPS Analysis

X-ray photoelectron spectroscopy with a Kα X-ray source (D/max-2500/PC) was utilized to determine the surface chemistry of the membranes.

#### 2.4.4. SEM Tests

Ultrafiltration membrane samples were extracted, cut into appropriate sizes, and put in clean Petri dishes. The sample was placed in a vacuum sensor and coated with a gold film to determine the conductivity of the sample. The coated samples were then mounted on the sample table of a scanning electron microscope (SEM) to take scanning electron microscope images of different areas.

#### 2.4.5. Water Flux and Salt Rejection

Water flux and salt rejection are important basic parameters of the membrane. The membrane performance was investigated using a lab permeation test under normal conditions. Before the membrane filtration tests, membranes need to be pre-pressurized with DI water for 5 min at 0.15 MPa to reach a steady state. Flux was introduced based on the following equation:

(1) $$WF=\frac{V}{A\times t}$$

where WF is the water flux (L/m^2^·h^−1^); V is the collected permeate water volume (L); A is the effective area of the membrane (m^2^, 28.7 cm^2^); and t is the permeation time (h). The WF characteristic of membranes was described according to the time needed to pass a specified volume of deionized water through the membrane.

This experiment measured the conductivity retention of the PES composite nanofiltration membrane in tap water before and after modification and measured the conductivity of the original solution and permeate.

#### 2.4.6. Molecular Weight Cutoff (MWCO)

Three experiments were carried out to test the performance of the membrane. The MWCO of membranes was plotted by measuring the rejection of PEG solutions with different molecular weights. The analytical method used for the PEG concentrations in the feed and permeated solutions was UV spectrophotometry. The rejection of the protein (R) was calculated by the following equation:

(2) $$R=1-\left(\frac{C_{p}}{C_{f}}\right)\times 100\%$$

where C_p_ and C_f_ are the permeate concentration and the feed concentration, respectively.

The membrane pore radius was measured on the basis of the PEG molecular weight and calculated as follows [33]:

(3) $$y=-5\times 10^{-8}\times x^{2}+5\times 10^{-4}x+0.3319$$

where y is the pore radius (nm), and x is the molecular weight of PEG (g/mol).

### 2.5. Performance Testing

Three experiments were carried out to test the performance of the membrane. The filtration experiments on the in-house-modified membranes were performed with an ultrafiltration cup, with prepared water in a laboratory (NO^3−^ 1.0 mg/L, NH^4+^ 0.5 mg/L, HA 3 mg/L). Equation (4) was used to evaluate the membrane efficiency in removing nitrogen pollutants from the solution.

(4) $$R\left(\%\right)=\left(1-\frac{C_{p}}{C_{f}}\right)\times 100$$

where C_f_ denotes concentration in the feed, and C_p_ denotes concentration in the permeate (mg/L). The concentrations of the feed and permeate solution were measured using UV-Vis absorption spectroscopy at a wavelength of 254 nm.

## 3. Results and Discussion

### 3.1. Effect of Mesoporous Silicon on the Hydrophilicity of PES Composite Membranes

The contact angle is analyzed by the contact angle of the liquid droplets on the solid surface when they form a thermodynamic equilibrium. It is an important index to measure the hydrophilicity of membranes, and it is also the main index to evaluate the wettability of solid surfaces. The effect of mesoporous silicon on the hydrophilicity of PES membranes will be discussed in detail in the effect of mesoporous silicon on antennae. The contact angle of NF270 nanofiltration membrane is high, reaching 84.95 degrees. As shown in Table 2, the measured values of the membrane contact angle were 0% Si/PES > NF270 > 5.0% Si/PES > 1.0% Si/PES > 0.5% Si/PES. Among them, the 0.5% Si/PES membrane has the best hydrophilicity with a contact angle of 67.65 degrees. Compared with the modified membrane, the contact angle of NF270 is smaller, which indicates that the modified membrane base liquid cannot reduce the contact angle. The decrease in the contact angle of other mesoporous silicon modified PES composite nanofiltration membranes is due to the addition of mesoporous silicon, which contains hydroxyl groups in its structure and enhances the hydrophilicity of the membrane surface [34,35]. This indicates that the addition of mesoporous silicon can enhance the hydrophilicity of the membrane surface, thus facilitating water transport. When more mesoporous silicon is added, the contact angle increases slightly, which is because mesoporous silicon may agglomerate on the surface of the membrane, which changes the uniformity of the membrane surface. However, this membrane is still more hydrophilic than the NF270 nanofiltration membrane.

### 3.2. Surface Charge of Mesoporous Silicon/PES Composite Membranes

**Figure 2:**
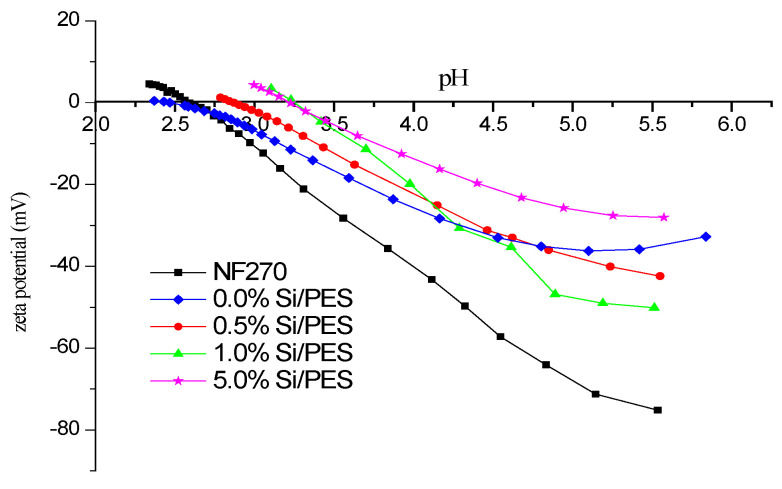
Zeta potential versus pH curves of the pure PES membrane and mesoporous silicon-modified PES membranes with different doping amounts.

The zeta potential of the membrane measured with NaCl as an electrolyte as a function of pH is shown in Figure 2. There was no significant difference between the modified membrane and the pure membrane. This may be because a small amount of modified material (mesoporous silicon) has some influence on the surface charge distribution of the membrane during the fabrication process. However, it was found that the surface charge of the NF270 composite film was different from that of the mesoporous silicon-modified PES composite film. The change in the surface charge of the membrane helps to improve the removal rate of nitrogen contaminants by the modified membrane, because the functional groups on the surface of the membrane improve the conductivity of the modified mesoporous silicon. Mesoporous silicon-modified polyethersulfone composite membranes can produce conductive materials, which may be because of the conductivity of mesoporous silicon. The conductance of PES composite membranes modified with 0.0 wt% mesoporous silicon has no obvious change. However, when 1.0 wt% mesoporous silicon or 5.0 wt% mesoporous silicon was modified onto polyethersulfone membranes, a significant contribution to the interfacial conductance was observed.

The isoelectric point (IEP) of the PES film with 0.0 wt% was higher than that of NF270. As shown in Table 3, mesoporous silicon changes at low pH. When the pH value is lower than the IEP value of the membrane, the surface of the membrane is positively charged, while, when the pH value is higher than the IEP value of the membrane, the surface of the membrane is negatively charged due to proton desorption and hydroxyl anion adsorption. Modified polyethersulfone membranes using 0.5 wt% mesoporous silicon were not significantly different from modified polyethersulfone membranes using 0.0 wt% mesoporous silicon, but the IEP values of modified polyethersulfone membranes using 0.5 wt% mesoporous silicon were biased towards higher pH values. The zeta potential of the composite membrane decreased with increasing pH, and it can be observed in Figure 2 that, the higher the pH, the more negative the zeta potential. Furthermore, it can be seen that an increase in the concentration of mesoporous silicon on the membrane surface leads to an increase in negative charges at pH 4–6. However, IEPs with 1.0 wt% or 5.0 wt% PES modified with mesoporous silicon did not seem to differ significantly. The surface of the modified Si/PES composite film has higher negative charges, which is due to the existence of functional groups on the surface. The increase in IEP values is due to the adsorption of anions Cl^−^ and OH^−^ in the solution [36,37]. The modified composite membrane has a negative charge and a stronger antifouling ability, which is due to the electrostatic interaction between HA molecules and the surface of the membrane. The negative charge of the modified composite membrane further reduced the adsorption of nitrogen contaminants on the modified membrane. The charge of the membrane has an important influence on the performance of the membrane, because the charge affects the electrostatic interaction between ions and charged molecules and the charge on the surface of the membrane.

### 3.3. XPS Analysis of Mesoporous Silicon/PES Composite Membranes

**Figure 3:**
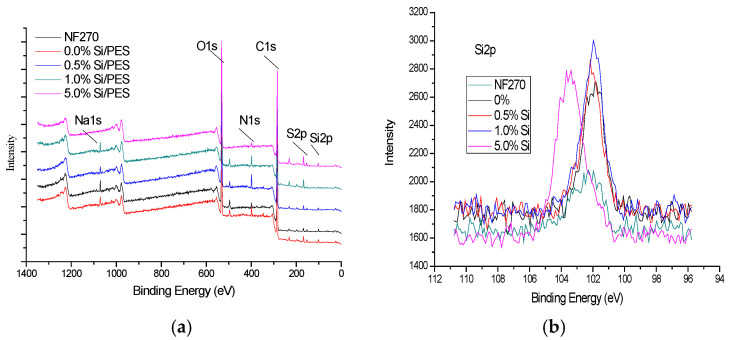
(**a**) XPS spectra of pure PES membrane and mesoporous silicon-modified PES membranes with different doping amounts; (**b**) Si2p spectra of pure PES membrane and mesoporous silicon-modified PES membranes with different doping amounts.

The chemical structure of the membrane surface was characterized by XPS spectra. Figure 3 shows the typical XPS spectra, and Table 4 lists the corresponding elemental composition values. It can be seen that both pure and modified membranes contain S, C, N, O, and Si elements. As can be seen from Figure 3b, the silicon peaks of the modified film and NF270 are significantly different depending on the content of the modified film. The Si2p absorption peak of the Si/PES membrane appears at 101 eV, which indicates that mesoporous Si has been successfully modified on the surface of the nanofiltration membrane. With an increase in the doping amount, the response value of the characteristic peak also increases, and the percentage of the specific element can be obtained from Table 2. The data show that, when the doping amount is lower than 0.5 wt%, the silicon content has a linear relationship with the response of the absorption peak. However, when the doping amount of mesoporous silicon is higher than 0.5 wt%, the linear relationship disappears due to the aggregation of mesoporous silicon at the bottom of the casting solution [38]. Compared with the standard XPS spectra, silicon atoms have characteristic absorption peaks at 99.3 eV, and tetravalent silicon has absorption peaks around 103.3 eV. It can be seen from Figure 3 that the Si/PES film has a strong absorption peak at 102 eV but no characteristic absorption peak at 99.3 eV, indicating that the mesoporous silicon modified on the film surface is tetravalent silicon and not silicon in the atomic state. The synthesis process of mesoporous silicon is the hydrolysis of silicic acid to form silica sol, and the silica sol and template self-assemble to form crystals. Therefore, mesoporous silicon mainly exists in the form of quadrivalent bonds.

### 3.4. SEM Test of Mesoporous Silicon/PES Composite Membranes

**Figure 4:**
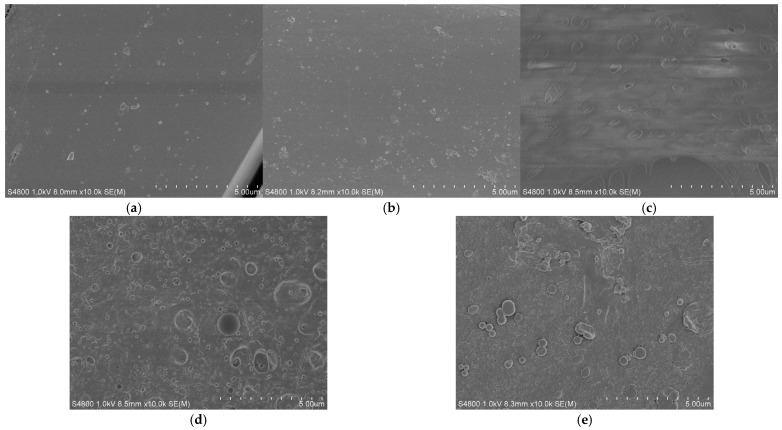
SEM of NF270 and the mesoporous silicon-modified ultrafiltration membrane with different doping amounts. (**a**) NF270, (**b**) 0.0%Si/PES, (**c**) 0.5%Si/PES, (**d**) 1.0%Si/PES, (**e**) 5.0%Si/PES.

In Figure 4, Figure 4a,b are the surface SEM maps of NF270 and 0.0% Si/PES, respectively, and the surface of the film is very smooth. Compared with Figure 4c–e, it can be seen that the surface of the modified film has granular mesoporous materials. As shown in Figure 4c,d, low-concentration mesoporous silicon can be dispersed in the coating solution, which plays a positive role in the construction of the film structure. It can be seen from the figures that the pores on the surface of the film increase significantly, and the distribution is relatively uniform. The promotion of mesoporous silicon on solvent and non-solvent diffusion reaches a relative balance, which can effectively promote the formation and dispersion of membrane pores, as shown in Figure 4c. However, due to the agglomeration of high-concentration mesoporous silicon in the coating solution, the distribution uniformity of pores in the figure becomes worse at this time. There are some large pores in some local areas, and some signs of unevenness may appear on the film surface.

### 3.5. Water Flux and Salt Rejection of Mesoporous Silicon/PES Composite Membranes

Through the water flux test results, we can see that the maximum water flux of 0.5% Si/PES membrane is 29.09 L/m^2^·h, which is nearly 73.88% higher than 16.73 L/m^2^·h of the pure PES membrane NF270 in the mesoporous silicon modified NF membrane. This is due to the different pore sizes and surface hydrophilicities of the membranes. The water flux of other modified membranes (e.g., 1.0% Si/PES membrane 56.16%) was higher than that of NF270. The reduced water flux of the 5.0% Si/PES membrane is due to the excessive doping of mesoporous silicon on the surface, which destroys the structure of the membrane, so it is difficult to form a uniform NF membrane structure [39]. The lowest water flux of 0.0% Si/PES membrane is due to low hydrophilicity. Generally speaking, the magnetic flux of all composite modified membranes containing mesoporous silicon is greater than that of pure PES membranes. Therefore, when the mass ratio of mesoporous silicon in the PES composite membranes is too high, the porosity of the membranes will decrease, and the flux will also decrease. Therefore, an improvement in water flux is conducive to an improvement in the treatment efficiency of the modified nanofiltration membrane, and the test results are consistent with the experimental results of the hydrophilicity of the membrane.

From the salt rejection data in Table 5, it can be seen that the modified membrane has a certain desalination rate on the basis of the original PES composite nanofiltration membrane, which is mainly due to the addition of mesoporous materials, which changes the surface structure of the nanofiltration membrane and the surface charge of the membrane. The desalination rate of the 0.5% Si-modified nanofiltration membranes is 32.61%, which is 44.05% higher than that of NF270. The desalination rate of the 1.0% Si- and 5.0% Si-modified membranes is 32.50% and 33.91% lower than that of NF270. For the purpose of this nanofiltration membrane modification, the main purpose is to intercept organic molecules and penetrate inorganic ions, so the decrease in desalination rate is more conducive to an improvement in the relative concentration of organic matter.

### 3.6. Pore Size Analysis of Mesoporous Silicon/PES Composite Membranes

**Figure 5:**
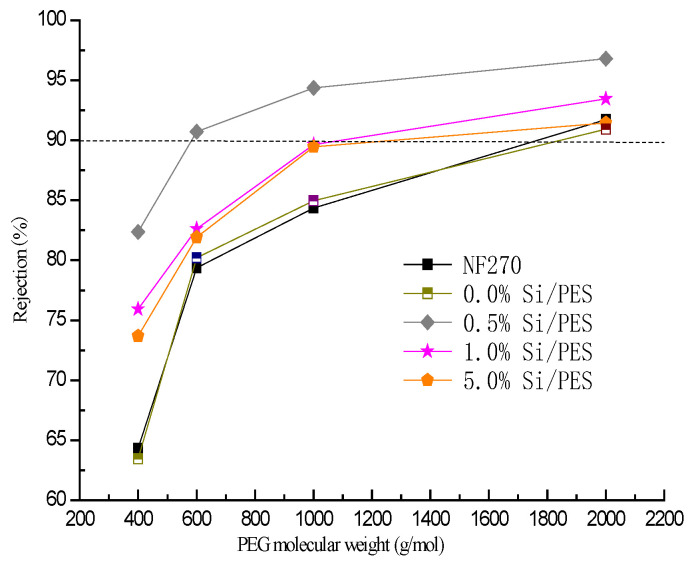
Rejection of the PEG solutions with different molecular weights (400, 600, 1000, 2000 Da) by pure PES membrane and mesoporous silicon-modified PES membranes with different doping amounts.

The structure of the membranes may be related to the pore size of the synthesized membranes. The pore size of the synthetic membrane was determined by the rejection of polyvinyl alcohol (PEG) (Figure 5). With an increase in PEG molecular weight, the PEG rejection rate of the NF film increased. When the PEG rejection of the membrane is greater than 90%, the molecular weight of the trapped material is the molecular weight of the membrane. Compared with NF270, the retention of 0.0% Si/PES, 1.0% Si/PES, 5.0% Si/PES was greater than 90% 2000 Da PEG, while the removal was lower than 90% 1000 Da PEG. According to the formula of membrane pore size (3), the membrane pore sizes of these PES films are 1.076 nm, 1.103 nm, 0.623 nm, 0.897 nm, respectively. The rejection of 0.5% Si/PES on 600 Da PEG was more than 90%, while the rejection of 400 Da PEG was less than 90%. The 0.5% Si/PES pore size of the modified film was calculated to be 0.581 nm. Compared with NF270, the pore size of the membrane is reduced, which is more conducive to the retention of inorganic molecules. The data show that doping of mesoporous silicon is very effective for reducing the pore size, and the pore size of PES composite nanofiltration membranes can be effectively controlled by changing the amount of mesoporous silicon, thereby improving the pore structure of the membranes.

### 3.7. Nanofiltration Performances of the PES Membranes

**Figure 6:**
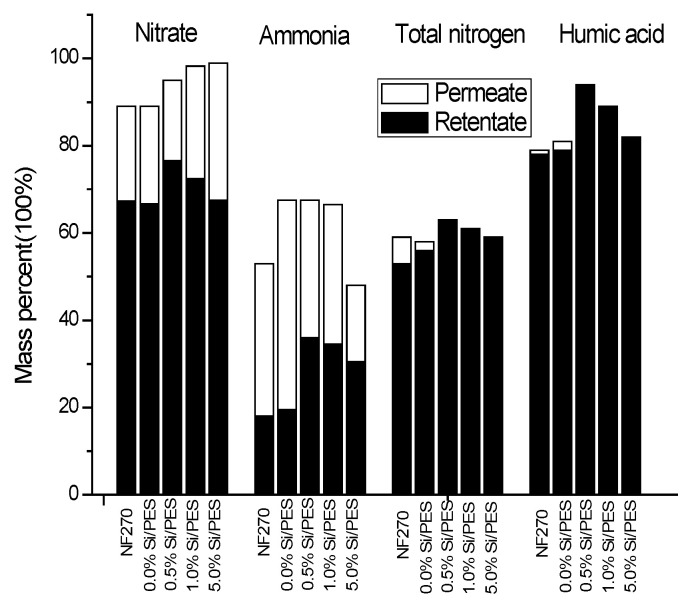
NF separation treatment for nitrogen pollutants with pure PES membrane and mesoporous silicon-modified PES membranes with different doping amounts.

**Figure 7:**
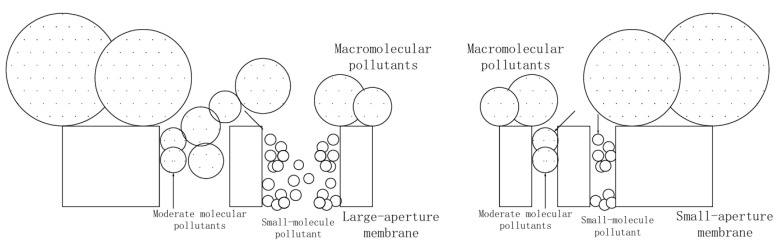
Schematic diagram of HA molecule diffusion in pores of membranes.

The concentration of ammonia nitrogen, nitrate nitrogen, and total nitrogen in osmotic and reflux solutions after nanofiltration treatment was analyzed at an operating pressure of 0.3 MPa. The results show that, on the surface of the film, as shown in Figure 6 and Table 6, the loss of nitrate is small, and the loss rate of ammonia nitrogen is more than 50%. The rejection rates of NF270, 0.0% Si/PES 0. 0% Si/PES, 0.5% Si/PES, 1.0% Si/PES, and 5.0% Si/PES to HA were 77.9%, 79.2%, 85.2%, 94.1%, and 81.9%, respectively. The main reason is that, when the pore size of the membrane is larger, contaminant molecules are more likely to enter the pore structure of the membrane, resulting in higher membrane loss of HA. The trend of membrane loss is consistent with the results of the PEG separation experiment and pore size analysis of the membrane in Section 3.6. HA is usually a mixture of materials of varying molecular sizes. Therefore, as shown in Figure 7, when the pore size of the membrane is larger, accumulation of HA is more likely to occur in the membrane channel.

This could be due to the decrease in pore restriction and the initial deposition of the HA colloids on the membrane surface which formed a filter layer on the membrane surface. The adsorption of HA molecules on the membrane surface obviously hindered the diffusion of inorganic nitrogen molecules. Therefore, the net driving force for the transmembrane transport of nitrogen pollutants decreases. Therefore, this work shows that the addition of mesoporous silicon to membranes enhances the antifouling capacity. The 0.5% Si/PES and 1.0% Si/PES composite membranes exhibited a higher flux rise of about 56.16–73. 88%. This observation is similar to the increase in membrane hydrophilicity and highly negatively charged surfaces (see Table 2 and Figure 2). Hydrophilic surfaces repel relatively hydrophilic HA molecules, thereby reducing the adsorption of HA and exacerbating membrane blockage. In addition, this indicates that the adsorption of HA on the modified membrane is weaker than that between HA and a pure PES composite membrane. In fact, composite membranes have a higher flux and a lower fouling propensity.

As can be seen from Figure 6, the rejection of ammonia nitrogen, nitrate nitrogen, and total nitrogen by mesoporous silicon-modified PES composite nanofiltration membrane in concentrated solution is 18.0–36.1%, 66.7–76.5%, and 53.2–62.9%, respectively. The high rate of nitrate interception is conducive to the interception of nitrogen-containing pollutants. However, the rejection rate of ammonia nitrogen is lower than that of nitrate due to the electrostatic action on the membrane surface. The rejection rate of NF 270 toward ammonia nitrogen was 18.0%, which was lower than that of the modified membrane. Therefore, the addition of mesoporous silicon is conducive to the interception of ammonia nitrogen and enhances the separation effect of inorganic nitrogen. The rejection rate of nitrate on NF270 was 66.7%, and the rejection rate of nitrate nitrogen on mesoporous silicon-modified PES composite nanofiltration membrane was higher. At the same time, the nitrate adsorption capacity of mesoporous silicon-modified membranes was lower than that of pure PES composite nanofiltration membranes. Therefore, the modified nanofiltration membranes have stronger anti-clogging ability. As can be seen from Figure 5, on the one hand, the high rejection of TDN is caused by the effective rejection of HA. On the other hand, it is mainly caused by the high rejection rate of nitrate nitrogen. The TDN retention rate of NF270 is 53.2%, while the TDN retention rate of the 0.5%Si/PES modified membrane which owned the best TDN retention rate is 62.9%. Compared with NF270, the modification of mesoporous silicon is beneficial to the retention of TDN.

The humic acid retention of the 0.5% Si/PES membrane was 16.1% higher than that of NF270. This means that different modified PES composite nanofiltration membranes are used to pretreat water samples, and the loss rate of humic acids is different. This is mainly due to the fact that, when the surface pore size of the membrane is large, contaminant molecules are more likely to enter the pore structure of the membrane, resulting in a higher membrane loss of HA.

## 4. Conclusions

This study utilized mesoporous silica to modulate the pore size distribution and electrostatic characteristics of the membrane matrix and successfully fabricated a PES composite membrane doped with mesoporous silica via phase inversion. Characterization revealed that the nanofiltration composite membrane possesses a highly negatively charged surface and a narrow pore size distribution. Coating mesoporous silica onto a microfiltration substrate regulated the interfacial polymerization process and the performance of the formed active layer. Owing to the balance of membrane structure, pore size, hydrophilicity, hydrogen-bond electrostatic interactions, and permeability, the modified membrane exhibited enhanced separation efficiency for nitrogen-containing pollutants, particularly humic acid (HA). Among all the fabricated membranes, the 0.5% Si/PES membrane demonstrated the strongest antifouling capability, achieving the highest water permeability (29.09 L/m^2^·h) and the smallest pore radius (0.581 nm). Concurrently, its superior antifouling properties facilitated the retention of HA adsorbed on the membrane surface. These results indicate that the morphology, hydrophilicity, rejection rate, and permeability of the modified membrane are correlated with the loading of mesoporous silica.

## Figures and Tables

**Table 1:** The composition of membrane casting solutions.

Membrane Type	PES (wt%)	Mesoporous Silicon Content (wt%)	DMAc
NF270	0	0.0	0
0.0%Si/PES	25	0.0	75
0.5% Si/PES	25	0.5	75
1.0% Si/PES	25	1.0	75
5.0% Si/PES	25	5.0	75

**Table 2:** Water contact angles of modified PES composite membrane surfaces.

Membrane Type	Contact Angle (°)
NF270	84.95
0.0%Si/PES	87.25
0.5% Si/PES	67.65
1.0% Si/PES	68.30
5.0% Si/PES	73.25

**Table 3:** IEP value of the pure PES membrane and mesoporous silicon-modified PES membranes with different doping amounts.

Sample	IEP
NF270	2.59
0.0%Si/PES	2.53
0.5% Si/PES	2.87
1.0% Si/PES	3.25
5.0% Si/PES	3.22

**Table 4:** Binding energies (BEs) of core electrons and elements type of pure PES membrane and mesoporous silicon-modified PES membranes with different doping amounts.

Samples	O1s		N1s		C1s		S2p		Si2p	
	BE (eV)	%	BE (eV)	%	BE (eV)	%	BE (eV)	%	BE (eV)	%
NF270	531.28	24.72	398.97	4.81	284.46	64.920	167.44	2.57	101.98	0.22
0.0%Si/PES	531.33	26.12	399.03	4.97	284.51	63.11	167.86	0.02	101.45	0.02
0.5% Si/PES	531.46	22.06	398.92	2.61	284.24	71.45	167.91	1.01	101.34	1.41
1.0% Si/PES	531.32	23.77	399.04	4.86	284.41	67.37	167.46	1.18	101.44	1.44
5.0% Si/PES	531.56	30.21	399.17	2.7	284.48	61.11	167.40	3.24	102.76	2.74

**Table 5:** Pure water flux and salt rejection of the pure PES membrane and mesoporous silicon-modified PES membranes with different doping amounts.

Sample	Pure Water Flux (L/m^2^ h)	Salt Rejection %
NF270	16.73	58.28
0.0%Si/PES	9.09	34.13
0.5% Si/PES	29.09	32.61
1.0% Si/PES	26.46	39.34
5.0% Si/PES	8.71	38.52

**Table 6:** NF separation effect for nitrogen pollutants with mesoporous silicon-modified PES membranes with different doping amounts.

Sample	NO^3−^ %		NH^4+^ %		TN %		HA %	
	Retentate	Permeate	Retentate	Permeate	Retentate	Permeate	Retentate	Permeate
NF270	67.3	21.7	18.0	35.2	53.2	5.9	78.1	1.1
0.0%Si/PES	66.7	22.3	19.5	48.3	56.1	2.0	78.9	2.1
0.5%Si/PES	76.5	18.5	36.1	31.5	62.9	0	94.2	0
1.0%Si/PES	72.4	25.8	34.5	32.0	61.0	0	88.7	0
5.0% Si/PES	67.4	31.5	30.5	17.5	58.9	0	82.0	0
